# Synergistic effect of cold atmospheric pressure plasma and free or liposomal doxorubicin on melanoma cells

**DOI:** 10.1038/s41598-021-94130-7

**Published:** 2021-07-20

**Authors:** Konstantina Pefani-Antimisiari, Dimitrios K. Athanasopoulos, Antonia Marazioti, Kyriakos Sklias, Maria Rodi, Anne-Lise de Lastic, Athanasia Mouzaki, Panagiotis Svarnas, Sophia G. Antimisiaris

**Affiliations:** 1 Pharmaceutical Technology Laboratory, Department of Pharmacy, University of Patras, 26504 Rion, Greece; 2 High Voltage Laboratory, Department of Electrical and Computer Engineering, University of Patras, 26504 Rion, Greece; 3 FORTH/ICE-ΗΤ, Institute of Chemical Engineering Sciences, 26504 Rion, Greece; 4 Laboratory of Immunohematology, Division of Hematology, Department of Internal Medicine, Medical School, University of Patras, 26500 Patras, Greece

**Keywords:** Cancer, Nanoscience and technology

## Abstract

The aim of the present study was to investigate combined effects of cold atmospheric plasma (CAP) and the chemotherapeutic drug doxorubicin (DOX) on murine and human melanoma cells, and normal cells. In addition to free drug, the combination of CAP with a liposomal drug (DOX-LIP) was also studied for the first time. Thiazolyl blue tetrazolium bromide (MTT) and Trypan Blue exclusion assays were used to evaluate cell viability; the mechanism of cell death was evaluated by flow cytometry. Combined treatment effects on the clonogenic capability of melanoma cells, was also tested with soft agar colony formation assay. Furthermore the effect of CAP on the cellular uptake of DOX or DOX-LIP was examined. Results showed a strong synergistic effect of CAP and DOX or DOX-LIP on selectively decreasing cell viability of melanoma cells. CAP accelerated the apoptotic effect of DOX (or DOX-LIP) and dramatically reduced the aggressiveness of melanoma cells, as the combination treatment significantly decreased their anchorage independent growth. Moreover, CAP did not result in increased cellular uptake of DOX under the present experimental conditions. In conclusion, CAP facilitates DOX cytotoxic effects on melanoma cells, and affects their metastatic potential by reducing their clonogenicity, as shown for the first time.

## Introduction

The use of cold atmospheric (-pressure) plasmas (CAPs) is increasingly being considered for various biomedical applications such as wound healing and blood coagulation^1–3^, sterilization and bacterial susceptibility^4–7^, treatment of cancer^6,8^, and various other pathologies^9–11^.

With regard to cancer treatment, various strategies using different types of CAPs have recently shown promising effects in cellular tumor models of breast cancer^12–14^ , cervical cancer^13–15^, liver cancer^16^, lung cancer^17^, skin cancer^18–22^, and other cancers^6,8^. Numerous therapeutic advantages of CAPs over treatments with conventional chemotherapeutic agents have been identified as a result of previous studies, the most important being probably the selective effect of cold plasma on normal and carcinoma cells^22–24^.

Exploration of possible methods to increase the anticancer effect of CAPs, by combinations with nanotechnologies^25–29^ and/or chemotherapeutic agents^30–36^ has recently been initiated. In several cases, different types of nanotechnologies such as iron oxide or gold nanoparticles have been found to strengthen the therapeutic effects of cold plasmas, and some potential mechanisms of action have been further explored or proposed.

Melanoma is a highly resistant and a very aggressive form of skin cancer accounting for only 1% of skin cancers but represents the majority of fatalities related with skin cancer. Early diagnosis and treatment is critical for prognosis/survival; primary melanoma has a 5-year survival rate of 99%, whereas metastatic melanoma only 27%^37,38^.

Doxorubicin DOX (or Adriamycin), is one of the most potent chemotherapeutic agents with significant therapeutic activity in many cancers. Due to its toxicity (especially its cardiotoxicity) its use is limited. The well-established ability of a liposomal DOX formulation (DOX-LIP), the first nanomedicine ever approved for human use (Doxil, Janssen Biotech Inc), to overcome the most serious side effects of DOX is a major advantage in the treatment of various cancers. Combinations of DOX-LIP with locoregional therapeutic approaches are currently being investigated as methods to further expand the therapeutic potential and applications of this nanomedicine^39^.

Herein, we aimed to investigate the combined (potentially synergistic) effect of CAP, in the form of dielectric-barrier discharge (DBD) based plasma jet, and DOX on melanoma cells (of murine and human origin). In addition to studying the therapeutic potential of CAP treatments by evaluation of their effect on melanoma cell proliferation (independently and combined with DOX), the mechanism of cell death was studied by Annexin V / Propidium iodide flow cytometry and trypan blue exclusion methods. Furthermore, the effect of CAP on the uptake of the drug by the cells was investigated.

Two novel aspects of the current study are that (i) in addition to free DOX, the potential of CAP to potentiate the therapeutic potential of a liposomal formulation of DOX (DOX-LIP) against melanoma cells was investigated, and (ii) the effect of separate and combined treatments with CAP and DOX (free and liposomal) on the clonogenic ability of melanoma cells was evaluated to understand the potential of CAP and/or combination treatments to affect the metastatic potential of melanoma cells.

## Results

### CAP distinct features

**Figure 1:**
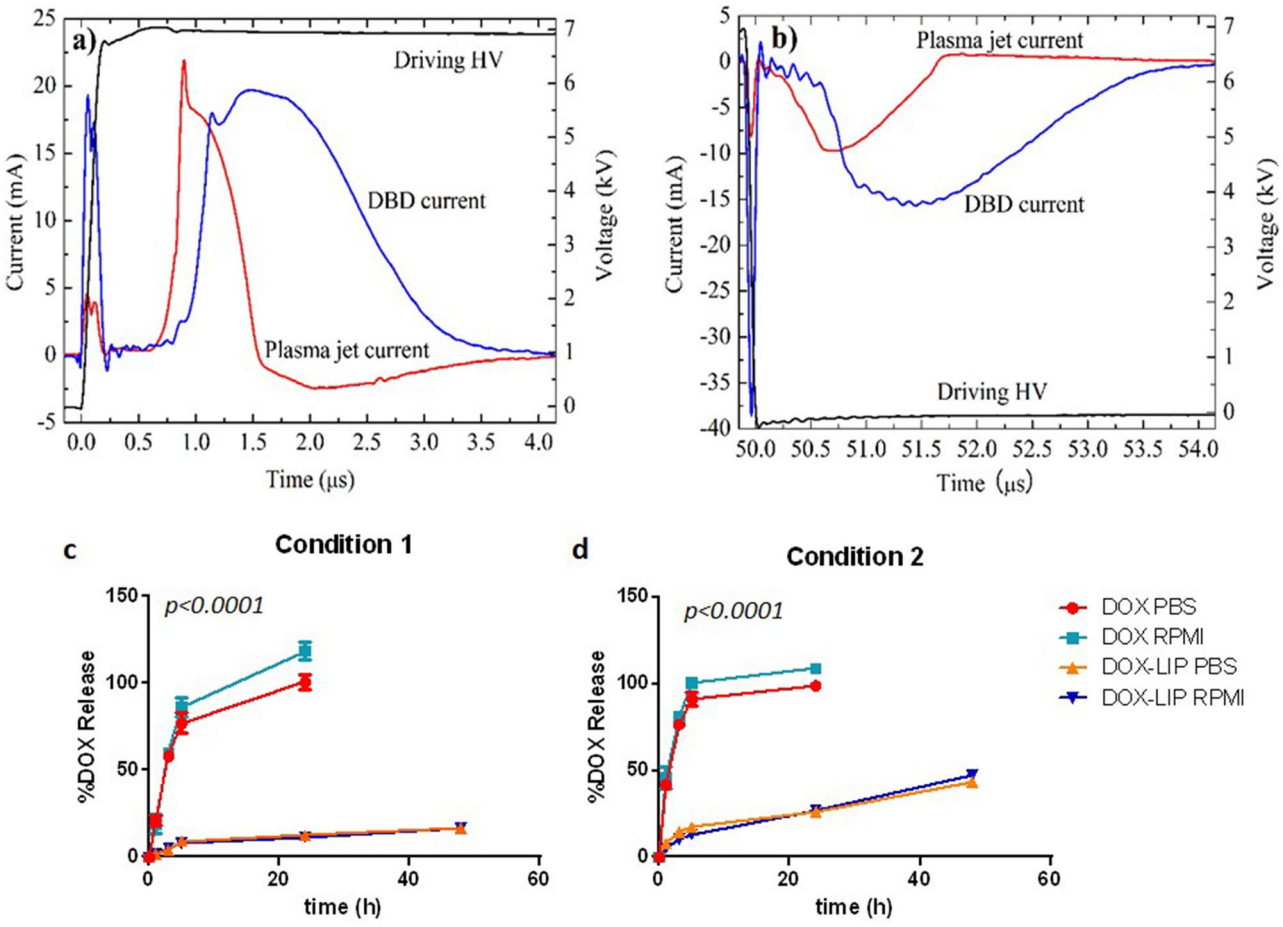
Representative waveforms of the driving pulsed high voltage, and the induced DBD and plasma jet currents, during: (**a**) The positive slope of the voltage pulse; (**b**) The negative slope of the voltage pulse. The records are carried out in situ, i.e. during specimen treatment. Release (% of total) of DOX from the liposomes during incubation for up to 48 h (at 37 °C), in PBS and RPMI 1640. (**c**) Release in Condition 1, 0.5 ml sample was taken out at each time point and (**d**) Release in Condition 2, 10 ml sample was taken out at each time point.

The driving voltage is provided by a laboratory made, square pulse, high voltage power supply, described in detail elsewhere^40^. The features of the voltage waveform are here adjusted as follows: pulse repetition rate 2 kHz, pulse amplitude + 7 kV, pulse duty cycle 10%. Typical oscillograms of the rising and falling slopes of this voltage are provided in Fig. 1a,b, respectively. In the same figures, both the DBD and the plasma jet current waveforms are given. These signals are recorded on a digital oscilloscope (LeCroy, WaveRunner 44Xi-A; 400 MHz—5 GSamples s^-1^) by means of broadband voltage and current probes (High voltage probe: PVM-4, North Star; DC—110 MHz. Current transformer: Pearson electronics 6585; 400 Hz–200 MHz).

Figure 1a,b presents typical waveforms of the driving voltage, and the induced DBD and plasma jet currents. All signals are recorded simultaneously in single shot mode and in-situ, i.e. when plasma plume impinges the fluid specimen. The latter is crucial for the reliable study of the above signals since notable modifications of them have been reported with respect to the free running plasma jet^41^. Based on these measurements, the electrical power delivered to the DBD and the plasma plume is estimated to be around 420 and 90 mW, respectively.

Regarding the physical properties and the chemical reactivity of such CAP jet systems, they have extensively been studied by our group in terms of free radical formation^42^, charged species production^43^, electro-hydrodynamic force and electric field development^44^, and thermal effects^45^ induced.

### Physicochemical characteristics of LIPs and DOX-LIPs

The physicochemical characteristics of the LIPs used in the following studies are presented in Table 1. As seen, LIP mean diameter (and polydispersity index) is slightly increased after DOX loading as well as the negative zeta potential of the vesicles. The polydispersity index of the LIPs is always lower than 0.33 for both liposome types, and as concluded by the detailed analysis of the intensity of peaks measured in each sample (see supplementary Table S1), despite the presence of a small percent of aggregated liposomes (< 2%), both samples are highly monodisperse. As anticipated the intensity (%) of the aggregated liposome peak, is much lower in the case of DOX-LIPs (compared to the empty LIPs, Table S1), which is reasonable due to their higher zeta-potential (Table 1). Finally, more than 95% DOX loading (DOX/lipid initial / DOX/lipid final) is achieved with the final drug/lipid ratio ranging between 0.18 and 0.20 mol/mol.

**Table 1:** Physicochemical properties of LIP and DOX-LIP with lipid composition DSPC/Chol/PEG (2:1:0.08 mol/mol/mol).

Sample	Mean diameter (nm)	PDI	Z-Potential (mV)	DOX-loading efficiency (% D/L)
Empty LIP	100.8 ± 2.6	0.264 ± 0.019	− 3.69 ± 0.42	–
DOX-LIP	110.8 ± 2.6	0.324 ± 0.069	− 7.63 ± 0.82	96.9 ± 1.8

Each value is the mean value calculated from three different preparations and the SD of each mean value is reported. Size values represent DLS intensity size calculations.

The reported physicochemical properties of empty LIP and DOX-LIP are in good agreement with the values reported before for similar LIP types^46,47^.

### Release of DOX

The time-courses of the release of DOX from DOX-LIP, during incubation for up to 48 h at 37 °C, under two experimental Conditions, 1 and 2, are presented in Fig. 1c,d, respectively. As expected free DOX, which was studied as a control, was totally released from the dialysis sacs during the first 5 h, under either Conditions tested. Oppositely, DOX release from DOX-LIPs was much slower, proving their high integrity. Under Condition 1 only 16% of DOX was release after 48 h whereas under Condition 2, 45% of DOX was released at the same time (Fig. 1c,d), indicating that most of the DOX was retained in the liposomes even under sink conditions (that do not apply in the cell studies). Importantly, the release of DOX from DOX-LIP was not affected by the presence of cell medium components such as serum (similar release in PBS and RPMI), proving that the liposomes used in the present study release DOX in a gradual and sustained manner, and that during the course of the LIP/cell interaction experiments described below, a very high fraction of the DOX is retained in the DOX-LIPs.

### Cytotoxicity, synergistic effect of DOX and CAP on B16F10 melanoma cells

**Figure 2:**
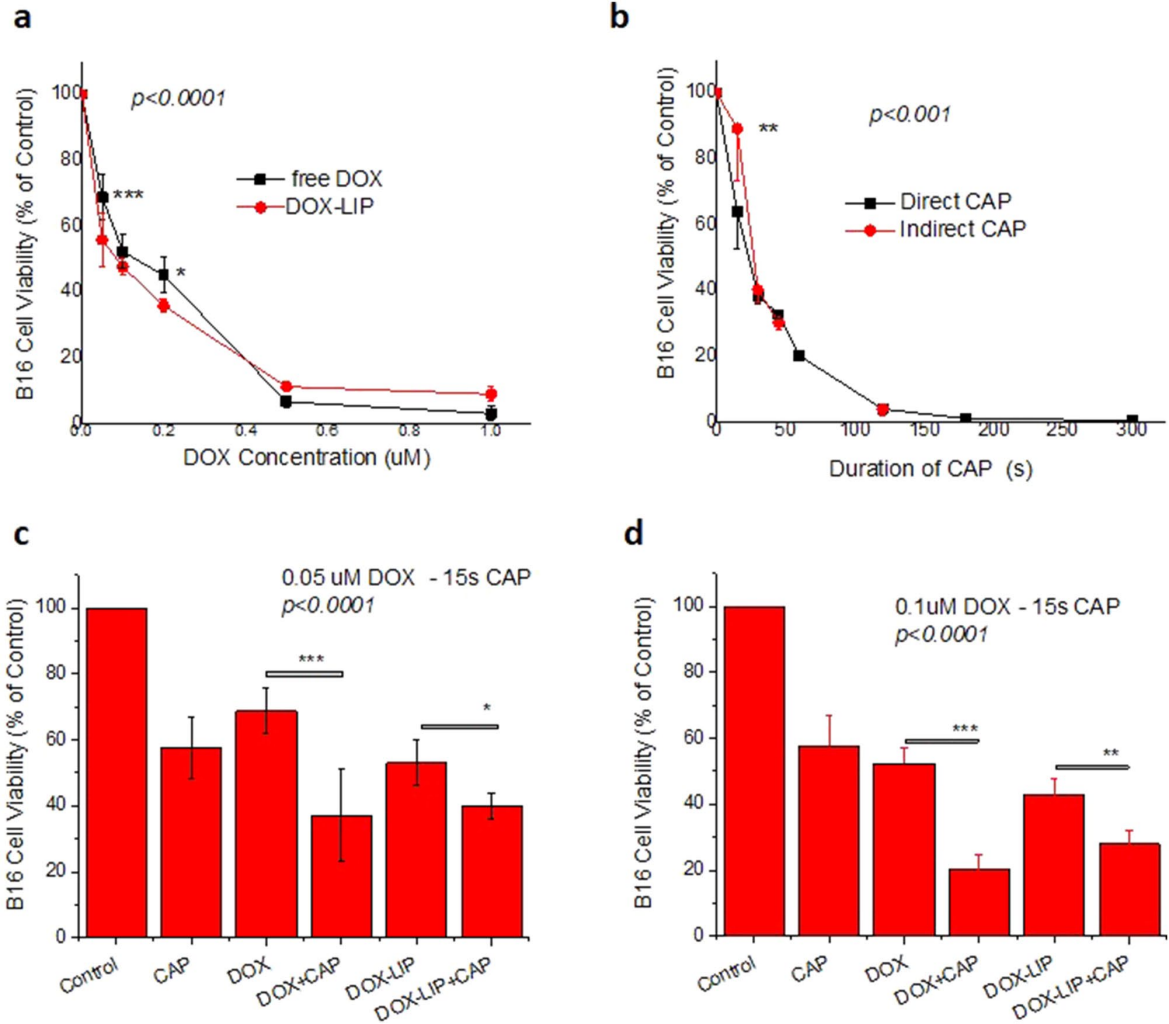
Effect of CAP and DOX or DOX-LIP on B16 cell viability. (**a**) Viability (% of control) of B16 cells after 48 h incubation in presence of various concentrations (0.05–1.00 μM) of DOX (free) and DOX-LIP. (**b**) Viability (% of control) of B16 cells after subjection to CAP for various time periods ranging between 15 and 300 s and incubation for 48 h. CAP was applied directly on the cells, or indirectly (on medium). Effect of CAP (15 s) and DOX or DOX-LIP on B16 cell viability, when applied separately or together, after 48 h of incubation with the cells (**c**) 0.05 μM DOX and (**d**) 0.1 μM DOX.

As shown in Fig. 2, treatment of B16F10 cells with increasing concentrations of DOX or DOX-LIP (Fig. 2a), as well as their subjection to CAP (for increasing periods of time) (Fig. 2b), results in a significant decrease in cell viability.

Comparing the effect of free DOX and DOX-LIP on B16F10 cell viability (Fig. 2a), it can be seen that DOX-LIPs are slightly more toxic to the cells, compared to free DOX (*p* < 0.05), at concentrations below 0.4μΜ. As can be seen from Fig. 2b direct subjection of CAP to B16F10 cells has similar effect as indirect CAP (treatment of medium with CAP and then incubation of the CAP-treated medium with cells for 48 h), with the only exception being the viabilities measured for 15 s of CAP treatment where the difference between direct and indirect CAP is significant. The later result may be due to the limited exposure time of cells/media under the plasma reactor, leading to an increased variability of the observed effect.

Figure 2c,d show the combined effect of CAP (15 s treatment) and DOX (as well as DOX-LIP), at DOX concentrations of 0.05 μM (Fig. 2c) and 0.1 μM (Fig. 2d); the results show a significant enhancement of the combined effect of CAP and DOX (free or liposomal), compared to the effects of the two treatments when applied independently. To understand whether there is synergism between the treatments CAP and DOX, the corresponding combination index values (CIs) were calculated according to a previously reported equation^46,48^. According to the latter, CI = (C1/Cm1) + (C2/Cm2), where, in the current case C1 and C2 are the dose of DOX (μM) and the duration of CAP (s), respectively, in combined administrations [CAP + DOX (or DOX-LIP)]; while Cm1 and Cm2 are the corresponding DOX dose and CAP duration (respectively), required in order to produce the same effect (% cell viability) when applied alone (just DOX or DOX-LIP and just CAP). CI values < 1, indicate synergism, equal to 1 additive effect, and > 1 antagonism^49,50^. All the CI values calculated from the available results for B16F10 cells (Fig. 2) are reported in Table 2. As can be seen, the results from all combined treatments of CAP (15 s) and DOX (or DOX-LIP) applied herein indicate a CAP/DOX synergistic effect, as the calculated CI values are much lower than 1 in all the cases.

**Figure 3:**
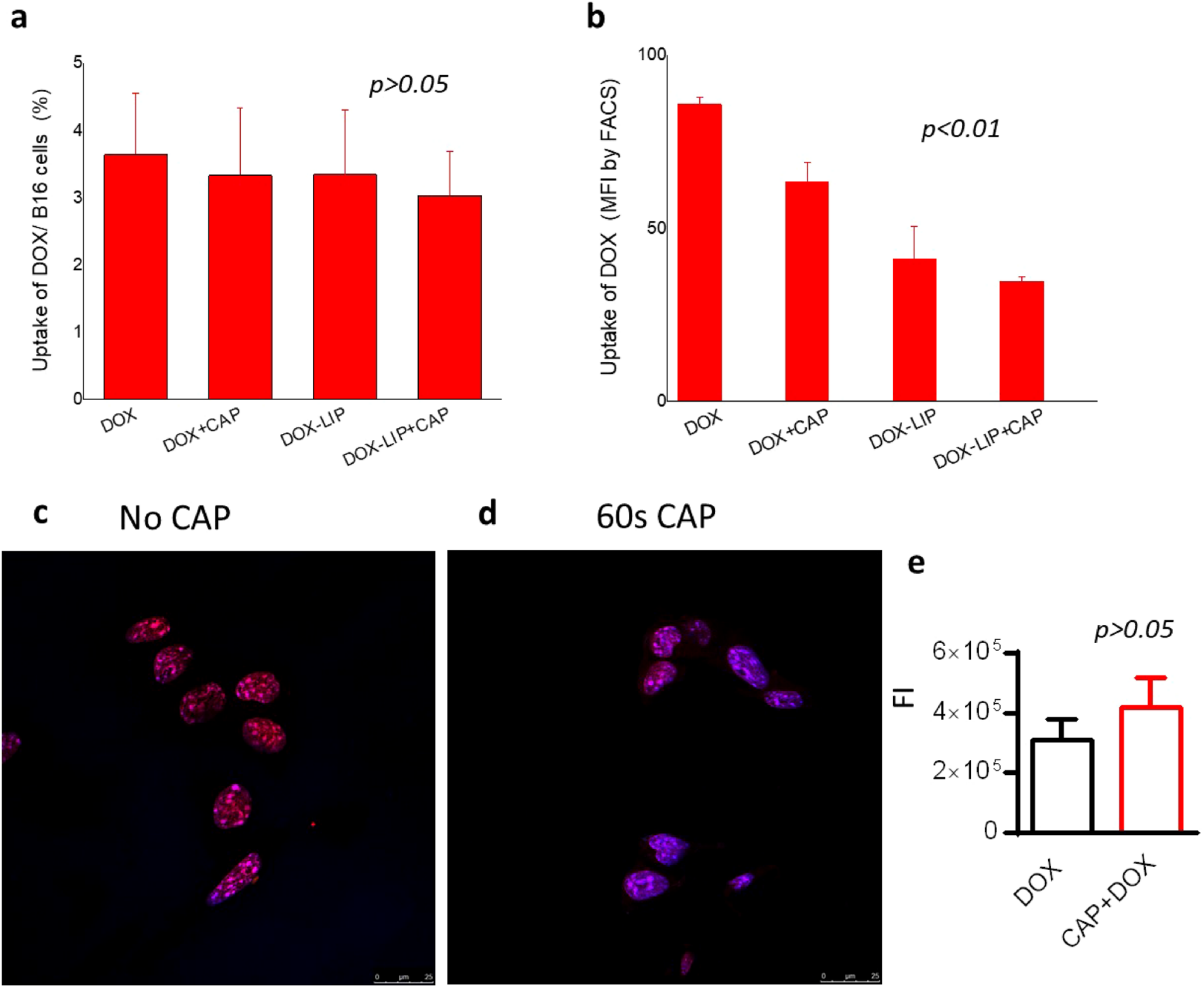
Effect of CAP on the uptake of DOX or DOX-LIP by B16 cells. (**a**) Effect of CAP (60 s) on the uptake of DOX or DOX-LIP by B16 cells after 4 h of co-incubation of cells with 10 μM DOX (free or liposomal) (**b**). Uptake of DOX by cells after 4 h co-incubation (same conditions as those applied in **a**), based on FACS measurement’s (Mean Fluorescence Intensity). (**c**,**d**) Representative LSCM micrographs of B16 cells following incubation with DOX (10 µM) only, or after subjection of the cells to CAP (for 60 s). (**e**) FI values calculated from micrographs by Image J.

**Table 2:** Combination Indices/indicators of synergistic effects between DOX (or DOX-LIP) and CAP on cell viability.

Cells	Conditions applied	Calculations	CI
B16F10	CAP + DOX (15 s & 0.05 μM)	0.05/0.25 + 15/35	0.628
	CAP + DOX (15 s & 0.1 μM)	0.1/0.35 + 15/60	0.536
	CAP + DOX-LIP (15 s & 0.05 μM)	0.05/0.15 + 15/35	0.762
	CAP + DOX-LIP (15 s & 0.1 μM)	0.1/0.3 + 15/50	0.630
SKMEL2	CAP + DOX (25 s & 0.5 μM)	0.5/5 + 25/^(>)^*60 = 0.1 + ^(<)^*0.41	^(<)^*0.51
HEK293	CAP + DOX (40 s & 0.1 μM)	0.1/^(>)^*1 + 40/^(>)^*60 = ^(<)^*0.100 + ^(<)^*0.66	^(<)^*0.766

*Absolute value was not available or could not be calculated.

### Effect of CAP on DOX uptake by B16F10 cells

The uptake of DOX (10 μM) by B16 cells was measured after 4 h or/and 24 h of co-incubation with different combinations of DOX, DOX-LIP, and CAP 60 s. Initially, it was verified that the cytotoxicity of DOX and CAP at 4 h was non-significant (see Supplementary Data; Fig. S1). Then, two methods were used: (i) Fluorescence Intensity (FI) measurements of cells and calculation of DOX uptake based on a calibration curve for DOX (Fig. 3a); and (ii) Flow Cytometry (FACS) Fluorescence measurements (mean FI) (Fig. 3b). As seen, in both cases there is no difference between the uptake values measured when DOX (or DOX-LIP) are incubated with cells alone, or when DOX and CAP are applied together (Fig. 3a,b). According to the FACS results (Fig. 3b), the interaction of DOX-LIPs with B16F10 cells was found to be significantly lower than free DOX. Co-treatment with CAP did not result in increased uptake of DOX by the cells; oppositely no statistical significant changes of uptake were noticed (for both free DOX and DOX-LIP), as also observed in the FI measurement results (Fig. 3a).

Morphological assessment of the nuclear distribution/localization of DOX in B16F10 cells, following co-incubation in absence and presence of CAP was carried out by confocal microscopy (LSCM), and representative micrographs for each case are presented in Fig. 3c (no CAP), and 3d (after 60 s CAP). As seen, in both cases DOX is taken up by all the nucleuses present in the micrograph frames, since no blue nucleuses are observed in any case, confirming the similar uptake percent measured. However, the color of the nucleuses present in the two micrographs is different, more purple and less red when cells were co-treated with CAP (compared to no CAP) revealing that the mechanism of DOX uptake by cells and nucleuses may be modulated by CAP. The FIs estimated from the micrographs (by Image J software 1.8.0) are in Fig. 3e; as seen, CAP treatment does not confer any significant difference in FI (*p* > 0.05).

### Effect of CAP on mechanism of cell death

**Figure 4:**
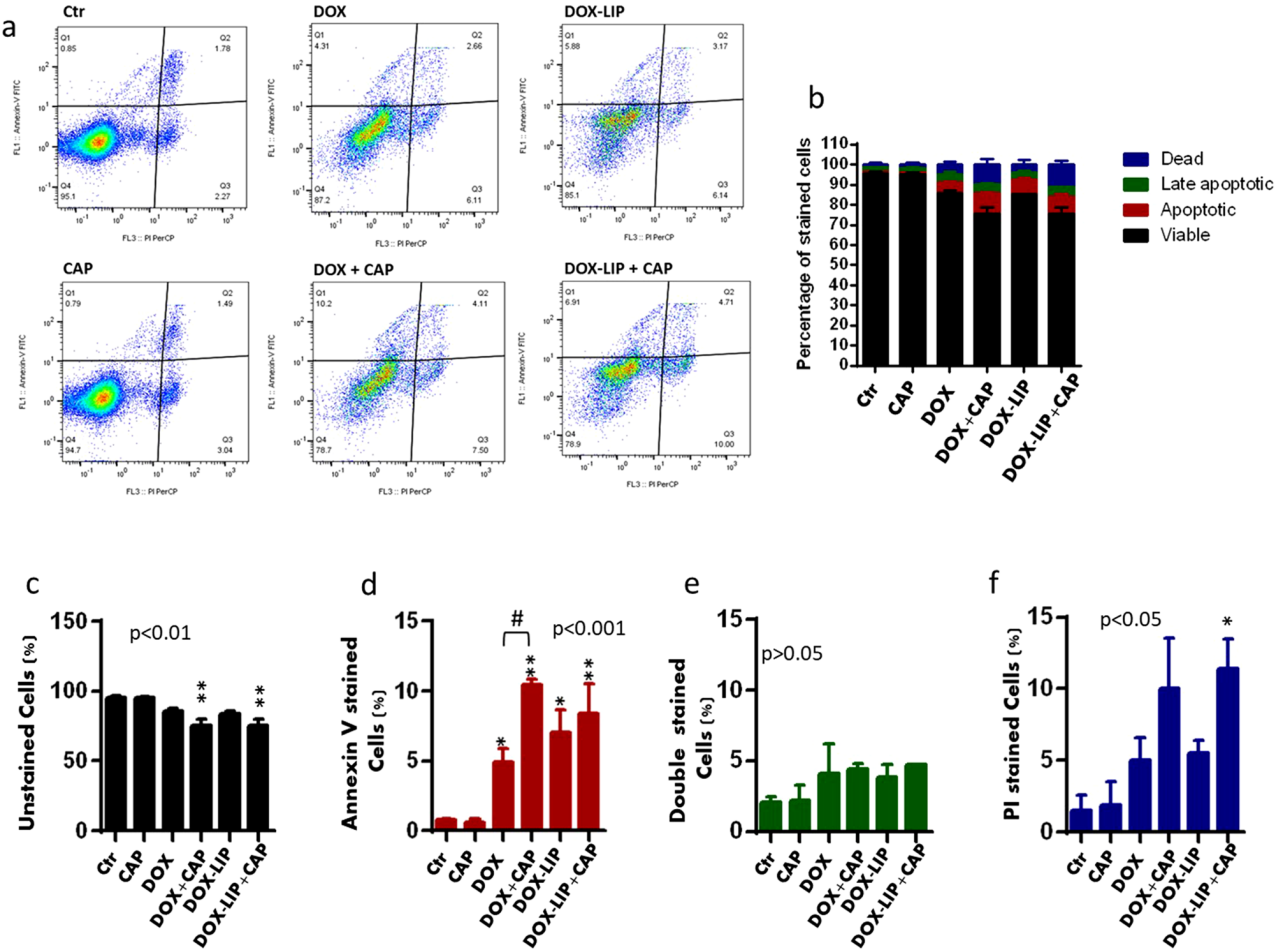
Apoptosis effect in B16F10 melanoma cells after treatment with CAP and/or DOX. (**a**) Flow cytometry with annexin V and PI dyes on B16 cells subjected to CAP (15 s) and/or treated with 0.1 μM DOX (free or liposomal) for 48 h. Dot plots of four-quadrant analysis Q4 involves the unstained, Q1 involves the early apoptotic (Annexin V positive), Q2 involves the late-apoptotic (double positive) and Q3 involves the dead (PI positive) cells. The number in each corner quadrant presents the percentage of cells in a typical experiment (n = 3). (**b**) Mean numbers from all flow cytometry experiments. (**c**–**f**) Separate analysis for each cellular subset. * is used when a group is statistical significant compared to ctr/CAP group and # is used when two different columns are compared, as indicated in each specific graph.

The results of flow cytometric analysis of apoptosis in B16F10 melanoma cells incubated with different combinations of 15 s-CAP, DOX, and DOX-LIP for 48 h after treatment are shown as a dot plot in Fig. 4a, where a four quadrant analysis was used. The number in each quadrant represents the percentage of cells in a typical experiment (n = 3), and the numbers from all experiments are summarized as a bar graph in Fig. 4b.

For clarity, a separate analysis was performed for each cellular subset as shown in Fig. 4c, for viable cells, Fig. 4d for apoptotic cells, Fig. 4e for late-apoptotic cells and Fig. 4f for dead cells. As seen, the various treatments applied have significant effects on the number of viable, apoptotic, and dead cells. In more detail, co-treatment with DOX (or DOX-LIP) and CAP resulted in a significant reduction in viable cells (compared to control), whereas single treatments with DOX (or DOX-LIP) or CAP resulted in a slight, non-significant decrease in the number of viable cells (Fig. 4c). As for the number of apoptotic cells (Fig. 4d) CAP alone had no effect, whereas treatment with DOX (free or liposomal) resulted in a significant increase in the number of apoptotic cells and, moreover, co-treatment with CAP accelerated the apoptotic effect of DOX in B16F10 cells; in the case of DOX-LIP, co-treatment with CAP did not significantly increased the number of apoptotic cells, compared with DOX-LIP alone,. Similarly, DOX (or DOX-LIP) increased the number of dead cells, but the differences were not significant due to high variability. Co-treatment with CAP resulted in almost twice the number of dead cells (compared to treatment with DOX/DOX-LIP alone), but the differences were statistically significant only for the DOX-LIP treatment (Fig. 4d).

The results of the trypan blue exclusion study performed on B16F10 melanoma cells 4 h, 24 h and 48 h after treatment with 15 s CAP and/or 0.1μΜ DOX (see Supplementary Data Fig. S2) also showed that the number of unstained cells was significantly reduced in the cells treated with DOX and the DOX + CAP (co-treatment), after 24 h and 48 h incubation, but not in the CAP (only) treated cells (Fig. S2). This fact is in consistence with the results of the apoptosis study (Fig. 4).

### Inhibition of colony formation by CAP and DOX (or DOX-LIP)

**Figure 5:**
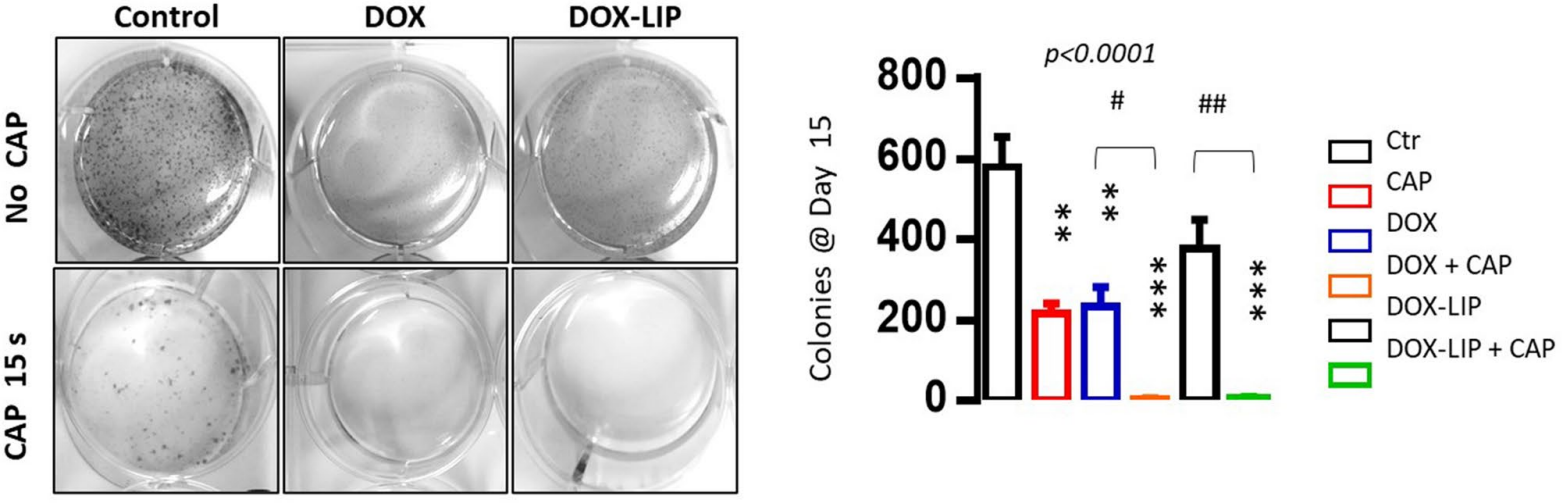
Effect of CAP exposure and/ or DOX treatment on colony formation capability by B16 melanoma cells. (**a**) Representative colonies are shown after 15 s CAP exposure or/and 0.1μΜ DOX treatment, free or liposomal. (**b**) Colony numbers counted after 15 d. Results are the mean ± standard deviation of three individual studies. *p < 0.05 or **p < 0.01 vs. control group under indicated culture conditions.

When cultured in soft agar, untreated B16F10 cells form compact spherical colonies that grow in size and number, as previously reported^49^. As shown in Fig. 5, 15s CAP treatment provided significant inhibition of colony formation, whereas DOX (free or liposomal), significantly decreased the number, as well as the size of the colonies, compared to the control. The combined treatment led to a synergistic additive inhibitory effect on the B16F10 mouse melanoma cell growth in soft agar, suggesting that CAP greatly enhances the inhibitory effect of the low DOX dose on colony formation. This is a very interesting result, because the clonogenic ability of cancer cells is considered to be highly related to their metastatic potential^51^.

### Studies on human melanoma (SKMEL2) and normal (HEK293) cells

To compare the interaction between mouse melanoma B16F10 cells and DOX (or DOX-LIP) and/or CAP, with other cell types, similar experiments were performed with human SKMEL2 melanoma cells and normal HEK293 cells.

**Figure 6:**
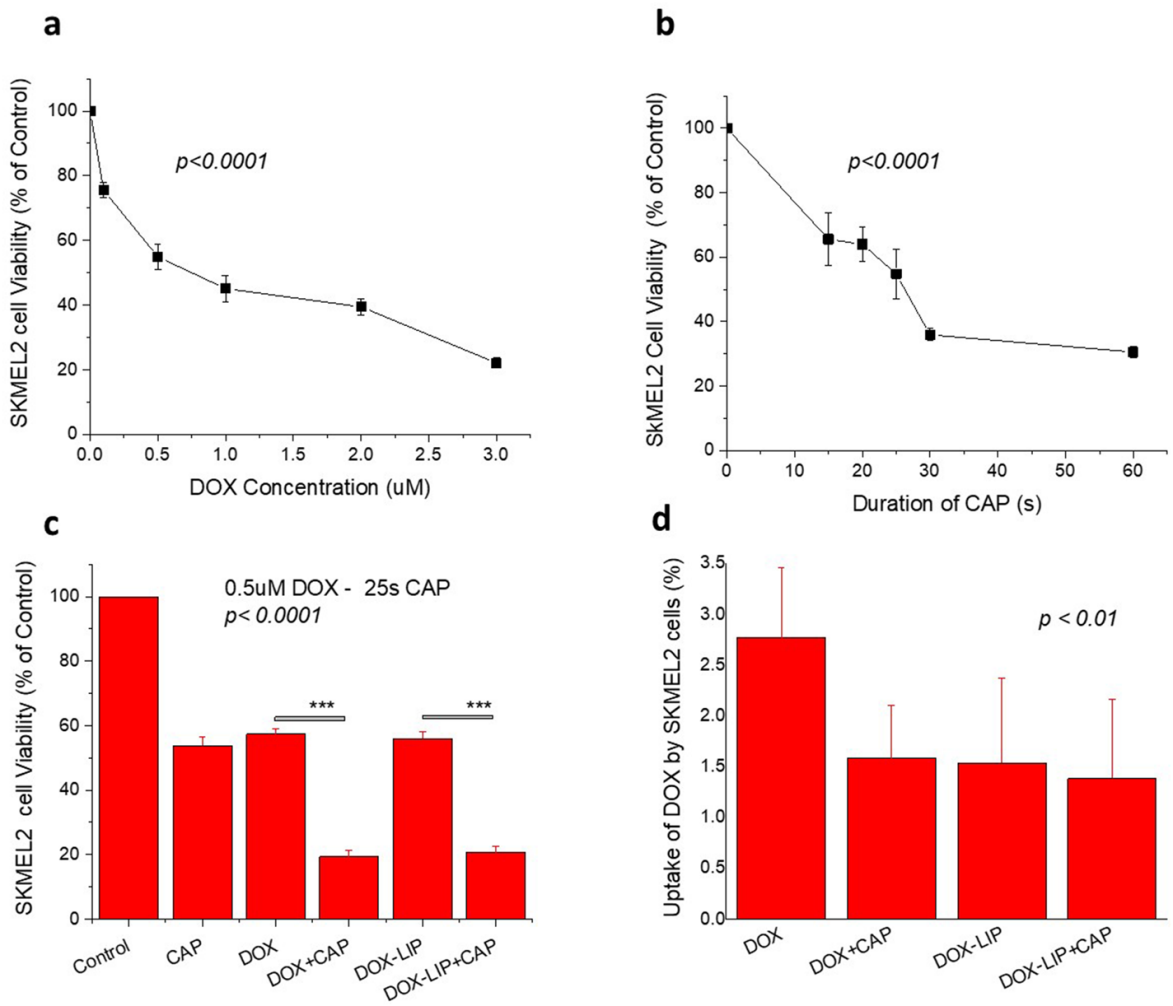
Effect of CAP and DOX or DOX-LIP on SKMEL2 cell viability. (**a**) Viability (% of control) of SKMEL2 human melanoma cells after 48 h incubation in presence of various concentrations (0.1–3.0 μM) of DOX (free); (**b**) Viability (% of control) of SKMEL2 cells after subjection to CAP for various time periods ranging between 15 and 60 s and incubation for 48 h. (**c**) Effect of CAP (25 s) and/or DOX (free of liposomal) treatment (0.5 μM DOX) on SKMEL2 cell viability, after 48 h of incubation with the cells. (**d**) Effect of CAP (60 s) on the uptake of DOX or DOX-LIP by SKMEL2 cells after 4 h of co-incubation of cells with 10 μM DOX (free or liposomal).

The results for SKMEL2 cells are shown in Fig. 6. Due to the higher resistance of SKMEL2 cells (compared to B16F10 cells) towards DOX and CAP, different treatment conditions were used. Figure 6a,b report the viability decrease of SKMEL2 cells after 48 h incubation in presence of increasing concentrations of DOX, or after increasing duration of CAP (15–60 s), respectively. Figure 6c depicts SKMEL2 cell viability (%) after treatment or not with CAP for 25 s and/or 0.5 μM DOX (or DOX-LIP) for 48 h incubation. As seen, individually applied CAP and DOX (or DOX-LIP) result in approximately 50% reductions in SKMEL2 viability. However, when applied together (CAP + DOX or DOX-LIPs) the cell viability values are reduced by more than 70%, suggesting a synergistic effect between CAP and DOX, as well between CAP and DOX-LIPs. The corresponding CI value is < 0.51 (Table 2), proving the synergistic effect of the combined treatment. The CI value for co-treatment of SKMEL2 cells with DOX-LIPs and CAP could not be calculated, since DOX-LIP effects on cell viability were not evaluated at different DOX concentrations. Nevertheless, the differences between the viability values realized after co-treatment (CAP + DOX-LIP) and after individual treatments (either CAP or DOX-LIP) are similar with the corresponding ones observed when free DOX was used.

As seen in Fig. 6d, treatment with CAP does not seem to enhance the uptake of DOX (or DOX-LIP) by SKMEL2 cells, as observed also in the case of B16F10 cells (Fig. 3a,b). In fact, the uptake of free DOX was reduced when CAP was co-applied to SKMEL2 cells. The uptake of DOX-LIP by SKMEL2 cells is lower compared to uptake of free DOX, but again it is not modulated by CAP.

**Figure 7:**
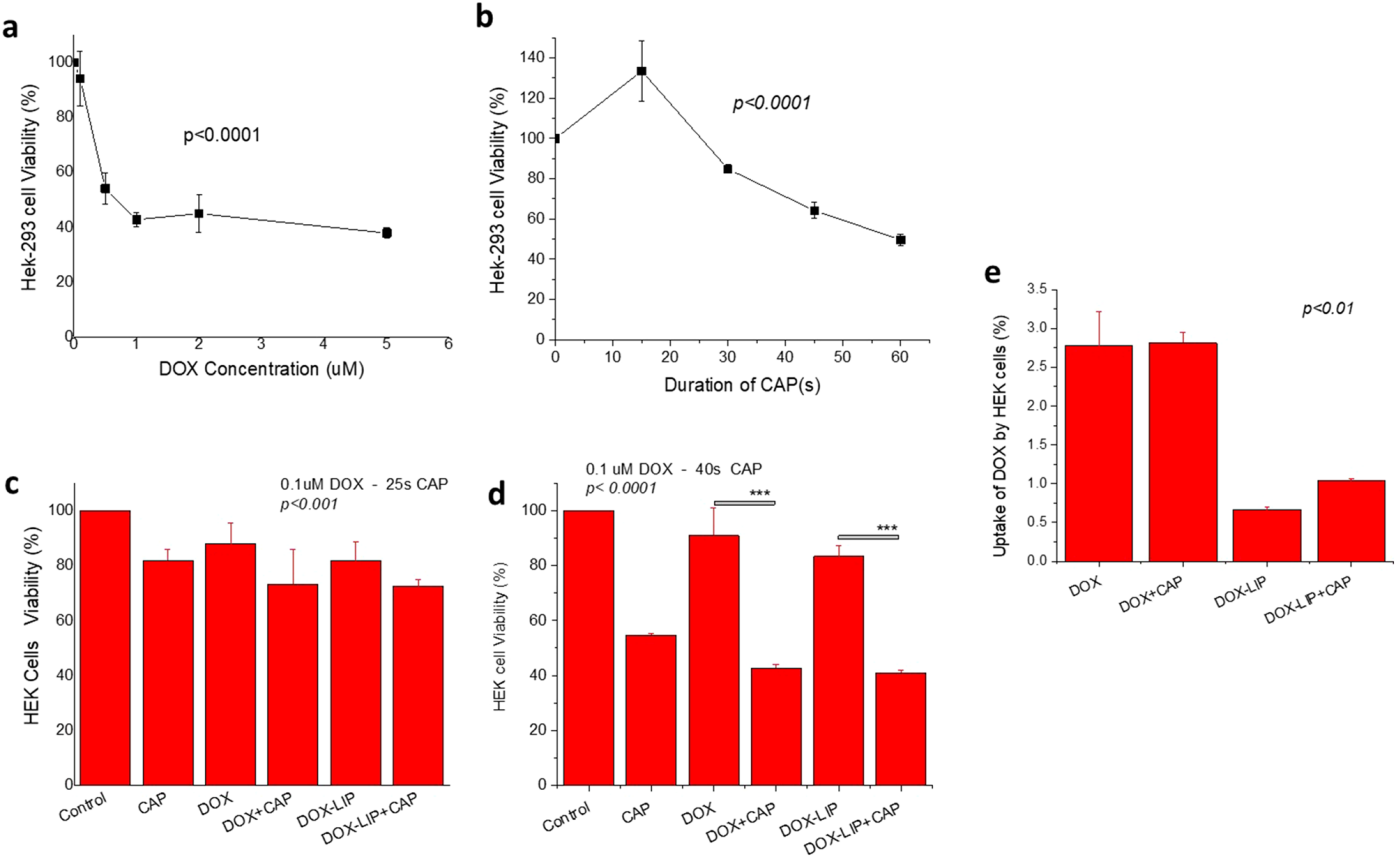
Effect of CAP and DOX or DOX-LIP on HEK293 cell viability. (**a**) Viability (% of control) of HEK293 cells after 48 h incubation in presence of various concentrations (0.1–5 μM) of DOX (free); (**b**) Viability (% of control) of HEK293 cells after subjection to CAP for various time periods ranging between 15 and 60 s and incubation for 48 h. (**c**,**d**) Effect of CAP (25 s in **c** and 40 s in **d**) and/or DOX (free of liposomal) treatment (0.1 μM DOX) on HEK293cell viability, after 48 h incubation (**e**) Effect of CAP (60 s) on the uptake of DOX or DOX-LIP by HEK293cells after 4 h of co-incubation of cells with 10 μM DOX (free or liposomal).

Concerning the effect of DOX (Fig. 7a) and CAP (Fig. 7b) on normal cells, HEK293 cells are more resistant to CAP compared with the human cancer cells SKMEL2 (Fig. 6b), and dramatically more resistant compared to the mouse melanoma cells B16F10 (Fig. 2b). In more detail, the corresponding viabilities following treatment with CAP for 60 s, is 49.6%, 30.6% and 20.2% for HEK293, SKMEL2 and B16F10 cells, respectively. Interestingly the viability of HEK293 cells was not reduced but instead increased when CAP was applied for 15 s (Fig. 7b).

Results of combined treatment of HEK293 cells with CAP and DOX or DOX-LIP are seen in Fig. 7c,d. In these cells CAP was applied for 25 s and not 15 s, since the later treatment increased the viability of HEK293 cells. As seen, co-treatment with DOX and 25 s CAP, did not result in significant augmentation of the DOX (or DOX-LIP) induced cytotoxicity (Fig. 7c), suggesting that HEK293 cells are much more resistant to CAP and DOX combination treatments, compared to B16F10 cells. Unfortunately we did not study the effect of combined CAP and DOX on HEK293 cells under the same conditions used for SKMEL2 cells, but when CAP was applied to HEK293 cells for 40 s together with 0.1 μM DOX (or DOX-LIP), their viability was significantly decreased (compared to individual treatments with DOX or CAP) (Fig. 7d). In fact, the CI value calculated for the latter experiments reveals a synergistic cytotoxic effect of the combined treatment (0.1 μM DOX and 40 s CAP; 48 h incubation) towards HEK293 cells (Table 2).

In respect to the effect of CAP on the uptake of DOX (or DOX-LIP) by HEK293 cells, as seen in Fig. 7e, CAP did not modulate uptake values (under the same conditions used to study the uptake of DOX by the cancer cell types). Of note, the uptake of DOX-LIP by HEK293 cells was 4 times lower than that of free DOX.

## Discussion

Herein we evaluated the effects of CAP jet on normal cells and melanoma cancer cells alone and in combination with a chemotherapeutic agent, DOX. In addition to the free drug, we also studied for the first time the combination of CAP with DOX-LIPs. Although some NPs have already been used for combined treatments with CAP before^25–29^, this is the first time that a synergistic effect of combined treatment with CAP and a liposomal drug has been studied.

Our results are in agreement with previous reports, regarding: (i) the selective effects of CAP on cancer versus normal cells; (ii) the comparative effect of direct and indirect application of CAP on cancer cells; (iii) the synergistic effect of CAP + DOX combinations; and (iv) the mechanism of the cytotoxicity of the combined treatments. Additionally we showed for the first time: (i) the synergistic effect of CAP + DOX-LIP combinations and (ii) the dramatic effect of combined CAP + DOX or CAP + DOX-LIP treatments on the clonogenicity of murine melanoma cells.

Human embryonic kidney cells (HEK293) were used as a model of normal cells. Selective cytotoxicity of cancer therapeutics toward cancer cells compared to normal cells is a highly desirable property for cancer treatments^22–24^. Selective effects of CAP against cancer cells compared to normal cells have been reported in many cases^12,15,22,23,52,53^, although it has been criticized that the conditions applied were not identical between the different cell-types studied or between the different studies, and that differences in experimental design may actually have a significant impact on CAP effects on cells^54,55^. In the current study, CAP exhibited significantly lower toxicity to HEK293 cells compared to cancer cells; all cells were studied under the same CAP reactor and identical culture conditions. An unexpected finding was the increase in HEK293 cell viability when treated with CAP for 15 s (Fig. 7b). Similar effects were recently reported in a study using B16F10 cells and fibroblasts (L929) where it was shown that CAP had the opposite effect on the proliferation of the two cell-types; fibroblast proliferation was increased by CAP^22^. Increased cell proliferation following 60 s CAP treatment was also reported for osteoblast-like MG63 (normal) cells, where the effect of CAP was attributed to increased expression of specific genes^56^. Although the related mechanisms of such effects of CAP on normal cell types have not been fully elucidated, our results confirm previous observations and emphasize the need for further exploitation of this interesting aspect of CAP.

Our results confirmed, through a separate set of experiments performed on B16F10 cells (Fig. 2b), that any aggravating effect of the current CAP setup is due solely to the effect of CAP on the cell culture medium. It is currently believed that CAP-treated fluids, termed "indirect CAP therapies", are of particular interest in oncology because they can kill cancer cells with similar efficiency to direct CAP, and thus have the advantage of avoiding the UV radiation and electric field associated toxicities (of direct CAP). Indirect CAP effects are attributed to the reactive oxygen and nitrogen species generated in the liquid media^23,56,57^.

As mentioned in the Results section, CAP-DOX synergism towards murine (Fig. 2c,d) and human melanoma cell (Fig. 6c) viabilities, is proven by corresponding CI values (Table 2). Interestingly, even at a very low DOX concentration (0.05 μM) that realizes minimal cytotoxicity towards B16F10 cells, co-treatment with CAP highly augments DOX cytotoxicity (Fig. 2c). Several reports on synergistic effects of CAP and cytotoxic drugs have been published^30–36^; in one study where B16 and human (SK-MEL-28) melanoma cells were tested, synergistic cytotoxicity of CAP with DOX and epirubicin and additive toxicity of oxaliplatin were reported^30^. The synergistic effects (with DOX) were attributed to enhanced intracellular accumulation of DOX via upregulation of the organic cationic transporter SLC22A16 as a result of CAP treatment. However, the conditions of the latter uptake experiment^30^, were different from the ones used in the current study (as detailed below).

Herein, the human melanoma cells were found to be more resistant to DOX and/or CAP, compared to the murine cells (Figs. 2a,b and 6). Higher DOX concentrations (0.5 μM compared to 0.1 μM) and longer CAP treatment (25 s compared to 15 s) were required for SKMEL2 cells (compared to B16F10) to attain similar effects (reduction of viability). Further studies are required to elucidate the different magnitude of responses. Nonetheless, similar differences between different cancer cells in their sensitivity to CAP have been previously reported. Compared to MCF-7 cells, MDA-MB-231 cells underwent a higher rate of apoptosis and a decreased proliferation rate upon CAP treatment^58^. The latter differences were attributed to potential CAP-mediated induction of epigenetic and cellular changes in a cell type-specific manner. In another case, CAP-activated medium inhibited proliferation and migration and induced apoptosis of triple negative breast cancer cells at a higher extent compared to other cell subtypes^59^. It was proposed that accelerated genome mutation rate and NF-κB signaling pathways of triple negative breast cancers made them more sensitive towards CAP^59^. The previous and current results suggest that careful screening of cells is required when considering potential cancer treatment applications of CAPs.

With respect to HEK293 cells, the combined treatment with 0.1 μM DOX + 40 s CAP demonstrated a synergistic cytotoxic effect; however the % viability values attained on normal cells by the combined treatments were not as low as the ones realized on the melanoma cells, which is in good agreement with the lower cytotoxic effect of CAP treatment (alone) towards HEK293 cells (compared to cancer cells).

As mentioned above, synergistic effects of CAP and drugs towards melanoma cells were attributed to enhanced intracellular accumulation of drugs^25,30,60^. In the current study, although synergistic effects were clearly observed, cellular uptake of DOX (by all cell types used) was not increased following CAP treatment: see Fig. 3 for B16F10 cells, 6d for SKMEL2 cells and 7e for HEK293 cells. However, looking at the conditions used for such experiments in other studies (in which CAP increased cellular uptake of NPs or drugs) we realize that they are very different from the conditions applying in the current studies^25,30,60^. For instance, CAP effect on Au NP uptake by U373MG Glioblastoma cells was evaluated after removal of culture medium from cells and then replacing it with fresh culture medium^25^. In another study, indirect CAP-enhanced cellular uptake of the fluorescent dye Dil was realized only after 3 or 5 days of treatment, but not before day 1^60^. Finally in the study already mentioned above^30^, cells and drug were initially incubated (for 6 h), then treated with CAP (30 s) and further incubated. In this regard, the fact that no increase in cellular uptake of DOX (or DOX-LIP) was observed in the presence of CAP might be related to the specific experimental conditions we used. At this point is should be pointed out that unfortunately, the potential cytotoxicity of co-treatment with CAP + DOX or (DOX-LIP) under identical conditions with those used in the uptake studies (60 s CAP and 10 µM DOX), was not evaluated (Fig. S1). Furthermore, due to high cytotoxicity, the cellular uptake experiments could not be performed under the conditions used in the studies showing CAP/DOX synergism (48 h).

Moreover, the current study proves for the first time that CAP supports the cancer-selective cytotoxic effect of DOX-LIP (in addition to free DOX). In fact, the results of the combined treatments with DOX-LIP and CAP also indicate that the two treatments have synergistic effect (CIs are < 1), towards both melanoma cell types studied; the decreases of cell viability realized towards normal cells were lower compared to cancer cells (as also seen when free DOX was used). As release experiments showed, DOX-LIP formulations present high integrity and lead to a more controlled and sustained release of the drug, rendering it safer towards normal, tumor—surrounding, cells.

In most of the previous studies in which NPs and CAP synergism has been explored, when metallic NPs, not loaded with any drug, were considered (such as gold NPs^25,28^, silver NPs^27^ and iron NPs^61,62^), CAP was found to enhance the activities acquired by NPs (when used) alone. In one report where curcumin-loaded NPs were used, CAP was applied as a practical approach to improve the aqueous solubility of curcumin during preparation of tri-phosphate chitosan nanoparticles TPP-NPs^26^. Curcumin TPP-NPs were not used in combination with CAP in the reported cell viability studies.

Considering the other reports, where drug-loaded NPs and CAP were used in combination, in one case fluorouracil (5-FU) loaded polylactic-co-glycolic acid (PLGA) NPs were used with CAP for synergistic inhibition of breast cancer cell growth. CAP induced down-regulation of metastasis-related gene expression (VEGF, MTDH, MMP9, and MMP2) and facilitated drug loaded NP uptake^35^. In another study, combined CAP and silymarin nanoemulsion treatment activated autophagy in G-361 cells by activating PI3K/mTOR and EGFR pathways, expressing autophagy-related transcription factors and genes^63^. Finally, paclitaxel-loaded PLGA NPs and CAP showed synergistic inhibition of A549 cells growth^64^. As concluded, the current results of the combined CAP and DOX-LIP treatment of melanoma cells are in line with the previous results where other types of nanoparticles or nano-formulations were used, proving that liposomal drugs could also demonstrate synergism with CAP.

Our results clearly demonstrate that the combined treatment with CAP and DOX (free or liposomal), further enhances the apoptotic potential of DOX against B16F10 cells, which is in line with previous reports demonstrating that DOX induces apoptosis in melanoma cells through p53 upregulation/accumulation^65^.

Finally, regarding the synergistic effect of DOX and CAP on clonogenicity and anchorage-independent growth of melanoma cells in soft agar, it should be noted that for cancer cells to undergo metastasis, they must have the ability to overcome anoikis and survive without cell-substrate interaction^66^. Anchorage-independent growth in soft agar has been shown to be one of the independent factors of the metastatic potential in cancer^67,68^. Herein we demonstrated for the first time that co-treatment with CAP (15 s) and DOX or DOX-LIP (0.1 μM) completely prevents the formation of B16F10 cell clones (Fig. 6). We did not find any relevant study on this topic in the literature, except for a recent publication reporting reduced expression of genes (VEGF, MTDH, MMP9, and MMP2) associated with cancer cell metastasis, due to the effect of cold plasma^35^. Our results suggest that CAP highly enhances the antimetastatic potential of DOX and DOX-LIP. This finding opens new insights into the potential applications of CAP for combination treatments of metastatic cancers and needs to be further explored in the future.

## Materials and methods

1,2-Distearoyl-sn-glycerol-3-phosphatidylcholine (DSPC) and 1,2-distearoyl-sn-glycerol-3-phosphoethanolamine-N-[methoxy(polyethyleneglycol)-2000] (DSPE-PEG2000 [PEG]), were purchased from Avanti Polar Lipids. Cholesterol (99%) (Chol), Doxorubicin hydrochloride, Trypan blue, agar, 3-(4,5-dimethylthiazol-2-yl)-2,5-diphenyltetrazolium bromide (MTT), Hoechst 33258, Triton X-100, Mowiol were purchased from Sigma-Aldrich. eBisocience Annexin V Apoptosis Detection Kit FITC was from Thermo Fisher Scientific. Ultrapure water was produced by a Direct-Q 3UV water purification system (Merck, Germany). RPMI1640, FBS, Trypsin–EDTA and all other cell culture solutions used were purchased from Gibco. Isopropanol, chloroform, methanol and all other chemicals used were of analytical quality and were purchased by Sigma-Aldrich. A rotor evaporator (Brucker), a bath sonicator (Branson), a microtip-probe sonicator (Sonics and Materials), and a hand held extruder with 400 nm, 200 nm and 100 nm pore-size polycarbonate membranes (Avestin), were used for liposome preparation. Fluorescence intensity (FI) of samples was measured by a Shimatzu RF-1501 spectrofluoremeter (Shimatzu, Kyoto, JP).

### Plasma reactor design and plasma treatment

**Figure 8:**
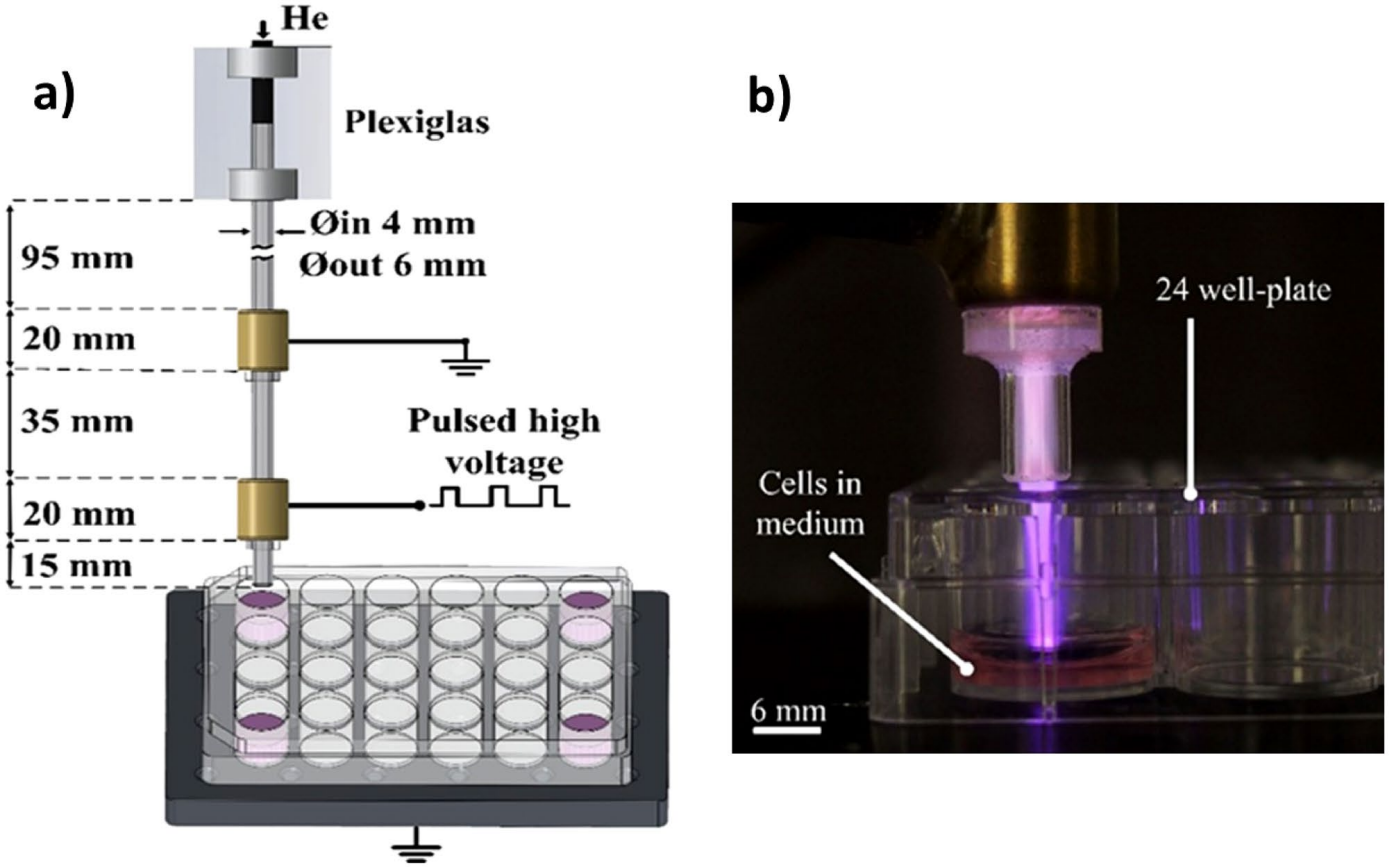
Design of the plasma jet reactor used for the present treatments. The electrode pair is driven by square high voltage pulses. (**a**) The specimens are placed in 24-well clear flat bottom plates hold on a grounded surface. (**b**) Typical image of the visible plasma plume in contact with the cell-medium specimen during the treatment.

The concept of the plasma reactor used for the specimen treatment is shown in Fig. 8a. The design refers to a dielectric-barrier discharge (DBD) based plasma jet of vertical orientation. It consists of a quartz tube and two external electrode rings made of brass. The geometry and the dimensions of these components are depicted in the same figure.

The lower electrode is the driven one and the upper electrode is directly grounded. The operating gas is high purity (99.999%) helium at a gas flow rate up to 5 slm. The latter is accurately controlled by a mass flow controller (Aalborg, GFC17) and it is here fixed at 2 slm.

The plasma jet was directed to the specimen wells, as Fig. 8b depicts. Thus, the plasma visible plume was in direct contact with the cell medium. The working distance (defined as that between the tube orifice and the well bottom) was fixed at 17 mm. Different treatment times were tested, i.e. the specimens were exposed to the cold atmospheric plasma (CAP) for periods between 15 and 300 s, depending on the particular study, as analyzed above.

### Liposome preparation

Liposomes (LIP) composed of DSPC/ Chol/PEG (2/1/0.08 mol/mol) were prepared by the thin film hydration method^69^. The thin lipid film was hydrated with ammonium sulfate buffer (120 mM, pH 5.5). After initial formation of the LIP dispersions, their size was reduced by probe sonication (Sonics & Materials). External ammonium sulfate was removed by ultracentrifugation (2 h at 40.000 rpm, Sorvall WX90 Ultra, Thermo Scientific) and liposomes were re-suspended in an isotonic PBS buffer (10 mM, pH 7.4).

### Preparation of DOX-LIP

DOX was loaded into LIPs using the remote active loading strategy, as recently described in detail^46^. For this, DOX (200 μg/ml) in PBS was added to the LIP dispersion (prepared as described above), and incubated for 1 h at 60 °C. Free DOX was removed by ultracentrifugation (1 h at 60.000 rpm). The Drug loading efficiency was then measured, as previously described in detail^47^.

### LIP size distribution and zeta potential measurements

The particle size distribution (mean hydrodynamic diameter and polydispersity index) of DOX-LIP dispersed in 10 mM PBS, pH 7.4 (at 0.4 mg/ml lipid) was measured by dynamic light scattering (DLS) (Malvern Nano-Zs, Malvern Instruments, Malvern, UK) at 25 °C and a 173° angle. Zeta Potential was measured in the same dispersions, at 25 °C, utilizing the Doppler electrophoresis technique.

### Release of DOX from DOX-LIPs

The integrity of DOX-LIPs was studied by measuring the release of DOX, during incubation in PBS or RPMI 1640 cell culture medium, for 48 h at 37 °C. Free DOX release under identical conditions was also measured for comparison. For this, 0.5 ml of sample (lipid concentration of 0.1 mg/ml) dispersed in PBS or RPMI was secured in dialysis tubing sacs (Servapor, with MW cutoff 14,000 Daltons), which were placed in capped test tubes containing 10 ml of PBS buffer, pH 7.4. The capped test tubes were then placed in a shaking incubator (Stuart Orbital Incubator) at 60 rpm and 37 °C. At specified time points (0, 1, 3, 5, 24, 48 h), 0.5 ml samples (Condition 1, simulating the conditions applying during the LIP/cell interaction studies), or 10 ml samples—the full amount of buffer—(Condition 2, sink conditions), were taken (sample volume was replaced with PBS) and DOX was quantified by measuring the sample FI (EX-485 nm/EM-590 nm) by a Shimadzu RF-Fluorescence Spectrophotometer.

### Cell culture

*C57BL/6* mouse B16F10 skin melanoma and human SKMEL2 skin melanoma cells were from the National Cancer Institute Tumor Repository (Frederick, MD). Human HEK293 embryonic kidney cells were from the American Type Culture Collection (Manassas, VA). All cell lines were cultured at 37 °C in 5% CO_2_-95% air using RPMI 1640 medium containing 10% fetal bovine serum, 2 mM L-glutamine, 1 mM pyruvate, 100 U/ml penicillin, and 100 mg/ml streptomycin.

### Cytotoxicity assay

Cell proliferation after cell treatment with cold atmospheric plasma (CAP) (15 s—300 s) and/or free DOX or DOX-LIP (0.1 μM—3 μΜ) was determined using the 3-(4,5-dimethylthiazol-2-yl)-2,5-diphenyltetrazolium bromide (MTT) assay^46^. For this, 25 × 10^3^ cells were plated onto 24-well plates. After 24 h, the cells were treated with CAP and/or DOX and/or DOX-LIP (which were added in the wells). The culture medium was then continuously retained without being replaced with fresh medium. After 48 h, MTT working solution (5 mM in PBS) was added in all wells. The plates were subsequently incubated for 2 h at 37 ^0^C in a 5% CO_2_ humidified incubator, and after that an equal volume of acidified isopropanol was added in each well for dissolution of the formazan crystals and measurement of absorbance at 620 nm on a Multiskan EX plate reader (Thermo, USA). Viable cells (%) were calculated based on the formula: (A-620[sample] − A-620[background])/(A-620[control] − A-620[background]) × 100, where A-620[control] is the OD-620 nm of untreated cells and A-620[background] the OD-620 nm of MTT without cells. Cytotoxicity experiments were carried out using different combinations of CAP and DOX or DOX-LIP, on the various cell types, in order to identify potential synergism between the two types of treatments (CAP and DOX).

### Trypan blue exclusion assay

48 h after treatment with DOX (0.1μΜ) and/or CAP (15 s), B16F10 and SKMEL2 cells were trypsinized with 0.25% trypsin/EDTA solution at 37 °C for 3 min and then neutralized with complete RPMI medium. After centrifugation, the cell pellet was re-suspended in PBS and cell suspension (10 µL) was mixed with 0.4% Trypan blue solution (10 µL). The stained cells (10 µL) were loaded into a countess cell counting chamber and the unstained (viable) as well as the stained (dead) cell numbers were counted using an automated cell counter (Invitrogen). The number of stained and unstained cells was then expressed as percentage of total cells counted.

### Cell uptake studies

For evaluation of the uptake of DOX by cells, free DOX or DOX-LIP (10μΜ) were incubated in the presence or absence of CAP (60 s), with confluent monolayer’s of B16F10, SKMEL2 or HEK cells, in full medium at 37 °C, for 4 h. After that, the medium was removed and the cells were washed twice with ice-cold PBS, detached from plates by scraping, re-suspended in 1 ml of PBS and assayed for FI (EX-485 nm/EM-590 nm), after cell lysis in 2% Triton X-100. Cell auto-fluorescence was always subtracted. The protein content of all samples was measured by the Bradford assay, and DOX uptake by cells was normalized to the cellular protein concentration of each sample.

DOX uptake by B16F10 cells after 4 h was also evaluated by flow cytometry under the same conditions described above. Flow cytometry was performed on a BD FACS Calibur flow cytometer (Becton Dickinson, Franklin Lakes, NJ). At least 20,000 events were acquired. DOX-positive cells were identified and their median fluorescent intensity (MFI) estimated using FlowJo v.10 software (Tree Star, Ashland, OR).

### Cell apoptosis/necrosis assay

The mechanism of cell death (apoptosis or necrosis) induced by CAP and/or DOX (or DOX-LIP) treatment, or their combination, was investigated on B16F10 cells [treated with DOX (or DOX-LIP) (0.1μΜ) following or not CAP (15 s) treatment]. After 48 h incubation cells were stained with Annexin V and Propidium iodide (PI) (eBioscience kit, Invitrogen) on a CyFlow ML flow cytometer (Partec, Munster, Germany). At least 20,000 events were acquired for each sample. Four-quadrant analysis of the results characterized the cells as viable (unstained), apoptotic (Annexin V positive), late-apoptotic (double positive) and dead (PI positive).The total cell numbers were expressed as % of total cell counts, using the FloMax software (Partec, Munster, Germany).

### Confocal fluorescence microscopy

B16F10 cells were grown on cover slips and incubated for 4 h with free DOX or DOX-LIP (5μΜ) in the presence/absence of CAP (60 s). The cells were then fixed in 4% paraformaldehyde for 10 min, stained with Hoechst 33,528 for 5 min and mounted on microscopy slides with Mowiol. Slides were observed using fluorescence microscopy on a SP5 confocal microscope (Leica, Heidelberg, Germany) to visualize the nuclear distribution/localization of DOX.

To quantify the cellular uptake of DOX, all imaging and processing settings were kept constant and the relative fluorescence intensities (FIs) were calculated with Image J (software version 1.8.0) according to the methodology contributed by Luke Hammond (QBI, University of Queensland, Australia) in the open lab book, as described in detail before^70^. https://theolb.readthedocs.io/en/latest/imaging/measuring-cell-fluorescence-using-imagej.html.

### Soft agar colony formation assay

It was examined whether CAP exposure and DOX treatment, alone and in combination, affected the ability of B16F10 melanoma cells to form colonies in soft agar. For evaluation of cell capability to form colonies on soft agar, 300 B16F10 cells were plated on 6 well plates in semi-solid agarose (0.7% w/v) in full culture medium, and treated with DOX (0.05μΜ) in the presence/absence of CAP (for 15 s), and were then incubated for 15d at 37 °C in a 5% CO2 humidified incubator. During the 15d incubation 1 ml of fresh culture medium was added to each well biweekly. After incubation, the cell medium was removed and 1.8 ml of PBS with 200 μl MTT working solution was added to each well. Plates were then dried, inverted, photographed and the numbers of colonies that have formed were counted.

### Statistical analysis

All results are expressed as mean value ± SD (SD stands for standard deviation) from at least three independent experiments. Most data were analyzed by using one-way ANOVA followed by Bonferroni post hoc test. Statistical significance for all comparisons was set at *p* = 0.05. When more factors were compared two-way ANOVA was performed. The significance of comparisons is presented on the graphs.

## Supplementary Information

Supplementary Information.

## Data Availability

All data generated or analyzed during this study are included in this published article.

## References

[1] Amini MR (2020). Beneficial effects of cold atmospheric plasma on inflammatory phase of diabetic foot ulcers; a randomized clinical trial. J. Diabetes Metab. Disord..

[2] Frescaline N (2020). Physical plasma therapy accelerates wound re-epithelialisation and enhances extracellular matrix formation in cutaneous skin grafts. J. Pathol..

[3] Nomura Y (2017). Investigation of blood coagulation effect of nonthermal multigas plasma jet in vitro and in vivo. J. Surg. Res..

[4] Guo L (2020). Microbial inactivation in model tissues treated by surface discharge plasma. J. Phys. D: Appl. Phys..

[5] İbiş F, Oflaz H, Ercan UK (2016). Biofilm inactivation and prevention on common implant material surfaces by nonthermal DBD plasma treatment. Plasma Med..

[6] Xu Z (2020). Applications of atmospheric pressure plasma in microbial inactivation and cancer therapy: A brief review. Plasma Sci. Technol..

[7] Svarnas P, Spiliopoulou A, Koutsoukos PG, Gazeli K, Anastassiou ED (2019). Acinetobacter baumannii deactivation by means of DBD-based helium plasma jet. Plasma.

[8] Elgendy AT, Abdallah T (2019). Cancer therapy system based on gold nanoparticle / cold plasma via stimulated singlet oxygen production. J. Phys.: Conf. Ser..

[9] Kim GC (2013). Dental applications of low-temperature nonthermal plasmas. Plasma Process. Polym..

[10] Laroussi M (2020). Cold plasma in medicine and healthcare: The new frontier in low temperature plasma applications. Front. Phys..

[11] Bansode AS (2014). Dielectric barrier discharge plasma for endodontic treatment. Commun. Comput. Inf. Sci..

[12] Pranda MA, Murugesan BJ, Knoll AJ, Oehrlein GS, Stroka KM (2020). Sensitivity of tumor versus normal cell migration and morphology to cold atmospheric plasma-treated media in varying culture conditions. Plasma Process. Polym..

[13] Vijayarangan V (2020). New insights on molecular internalization and drug delivery following plasma jet exposures. Int. J. Pharm..

[14] Jezeh MA, Tayebi T, Khani MR, Niknejad H, Shokri B (2020). Direct cold atmospheric plasma and plasma-activated medium effects on breast and cervix cancer cells. Plasma Process. Polym..

[15] Feil L (2020). Cancer-selective treatment of cancerous and non-cancerous human cervical cell models by a non-thermally operated electrosurgical argon plasma device. Cancers.

[16] Vaquero J (2020). Cold-atmospheric plasma induces tumor cell death in preclinical in vivo and in vitro models of human cholangiocarcinoma. Cancers.

[17] Amini M, Ghanavi J, Farnia P, Karimi M, Ghomi H (2020). In vitro antiproliferative activity of cold atmospheric plasma on small-cell lung carcinoma. Biomed. Biotechnol. Res. J..

[18] Terefinko D (2021). Biological effects of cold atmospheric pressure plasma on skin cancer. Plasma Chem. Plasma Process..

[19] Clemen R, Heirman P, Lin A, Bogaerts A, Bekeschus S (2020). Physical plasma-treated skin cancer cells amplify tumor cytotoxicity of human natural killer (NK) cells. Cancers.

[20] Pasqual-Melo G (2020). Plasma treatment limits cutaneous squamous cell carcinoma development in vitro and in vivo. Cancers.

[21] Rafiei A (2020). Inhibition of murine melanoma tumor growth in vitro and in vivo using an argon-based plasma jet. Clin. Plasma Med..

[22] Feng Z (2019). Cytotoxicity to melanoma and proliferation to fibroblasts of cold plasma treated solutions with removal of hydrogen peroxide and superoxide anion. IEEE Trans. Plasma Sci..

[23] Kim SJ, Chung TH (2016). Cold atmospheric plasma jet-generated RONS and their selective effects on normal and carcinoma cells. Sci. Rep..

[24] Yan D, Sherman JH, Keidar M (2017). Cold atmospheric plasma, a novel promising anti-cancer treatment modality. Oncotarget.

[25] He Z (2020). Cold atmospheric plasma stimulates clathrin-dependent endocytosis to repair oxidised membrane and enhance uptake of nanomaterial in glioblastoma multiforme cells. Sci. Rep..

[26] Sadoughi A, Irani S, Bagheri-Khoulenjani S, Atyabi SM, Olov N (2020). Cold atmospheric plasma modification of curcumin loaded in tri-phosphate chitosan nanoparticles enhanced breast cancer cells apoptosis. Polym. Adv. Technol..

[27] Manaloto E (2020). Cold atmospheric plasma induces silver nanoparticle uptake, oxidative dissolution and enhanced cytotoxicity in glioblastoma multiforme cells. Arch. Biochem. Biophys..

[28] Jawaid P (2020). Small size gold nanoparticles enhance apoptosis-induced by cold atmospheric plasma via depletion of intracellular GSH and modification of oxidative stress. Cell Death Discov..

[29] Pasqual-Melo G, Gandhirajan RK, Stoffels I, Bekeschus S (2018). Targeting malignant melanoma with physical plasmas. Clin. Plasma Med..

[30] Sagwal SK, Pasqual-Melo G, Bodnar Y, Gandhirajan RK, Bekeschus S (2018). Combination of chemotherapy and physical plasma elicits melanoma cell death via upregulation of SLC22A16. Cell Death Dis..

[31] Akopdzhanov AG (2019). The cytotoxicity of cold atmospheric plasma against HeLa cancer cells and its modification with pharmaceutical substances. Biophysics (Russian Federation).

[32] Gjika E (2020). Combination therapy of cold atmospheric plasma (CAP) with temozolomide in the treatment of U87MG glioblastoma cells. Sci. Rep..

[33] Lee C-M, Jeong Y-I, Kook M-S, Kim B-H (2020). Combinatorial effect of cold atmosphere plasma (Cap) and the anticancer drug cisplatin on oral squamous cell cancer therapy. Int. J. Mol. Sci..

[34] Daeschlein G (2013). Comparison between cold plasma, electrochemotherapy and combined therapy in a melanoma mouse model. Exp. Dermatol..

[35] Zhu W (2013). Synergistic effect of cold atmospheric plasma and drug loaded core–shell nanoparticles on inhibiting breast cancer cell growth. Sci. Rep..

[36] Xu D (2019). Plasma enhance drug sensitivity to bortezomib by inhibition of cyp1a1 in myeloma cells. Transl. Cancer Res..

[37] Siegel RL, Miller KD, Fuchs HE, Jemal A (2021). Cancer statistics. CA Cancer J. Clin..

[38] Eddy K, Shah R, Chen S (2021). Decoding melanoma development and progression: Identification of therapeutic vulnerabilities. Front Oncol..

[39] Gabizon AA, Patil Y, La-Beck NM (2016). New insights and evolving role of pegylated liposomal doxorubicin in cancer therapy. Drug Resist Updat..

[40] Koliadimas A, Apostolopoulos D, Svarnas P, Sklias K, Athanasopoulos D, Mitronikas E (2019). A microcontroller based modular pulsed H.V. power supply: Design, implementation and tests on DBD-based plasmas. IEEE Trans. Plasma Sci..

[41] Athanasopoulos, D. K. & Svarnas, P. Power alteration in a DBD-based plasma-jet system due to its interaction with aqueous solutions. *XXXIV International Conference on Phenomena in Ionized Gases* (ICPIG), (14-19.07.2019, Sapporo, Japan), PO16PM008 (2019).

[42] Gazeli K, Svarnas P, Held B, Marlin L, Clément F (2015). Possibility of controlling the chemical pattern of He and Ar “guided streamers” by means of N_2_ or O_2_ additives. J. Appl. Phys..

[43] Gazeli K, Svarnas P, Vafeas P, Papadopoulos PK, Gkelios A, Clément F (2013). Investigation on streamers propagating into a helium jet in air at atmospheric pressure: Εlectrical and optical emission analysis. J. Appl. Phys..

[44] Papadopoulos P (2019). Generic residual charge based model for the interpretation of the electro-hydrodynamic effect in cold atmospheric pressure plasmas. Plasmas Sources Sci. Technol..

[45] Svarnas P (2018). Parametric study of thermal effects in a capillary dielectric-barrier discharge related to plasma jet production: Experiments and numerical modelling. J. Appl. Phys..

[46] Zagana P, Mourtas S, Basta A, Antimisiaris SG (2020). Preparation, physicochemical properties, and in vitro toxicity towards cancer cells of novel types of arsonoliposomes. Pharmaceutics.

[47] Skouras A, Papadia K, Mourtas S, Klepetsanis P, Antimisiaris SG (2018). Multifunctional doxorubicin-loaded magnetoliposomes with active and magnetic targeting properties. Eur. J. Pharm. Sci..

[48] Batra H, Pawar S, Bahl D (2019). Curcumin in combination with anti-cancer drugs: A nanomedicine review. Pharm. Res..

[49] Chou T-C, Talalay P (1984). Quantitative analysis of dose-effect relationships: The combined effects of multiple drugs or enzyme inhibitors. Adv. Enzyme Regul..

[50] Jin J, Wang F-P, Wei H, Liu G (2005). Reversal of multidrug resistance of cancer through inhibition of P-glycoprotein by 5-bromotetrandrine. Cancer Chemother. Pharmacol..

[51] Agalioti T (2017). Mutant KRAS promotes malignant pleural effusion formation. Nat. Commun..

[52] Alimohammadi M (2020). Cold atmospheric plasma is a potent tool to improve chemotherapy in melanoma in vitro and in vivo. Biomolecules.

[53] Bisag A (2020). Plasma activated ringer’s lactate solution displays a selective cytotoxic effect on ovarian cancer cells. Cancers.

[54] Yan D (2015). Toward understanding the selective anticancer capacity of cold atmospheric plasma—A model based on aquaporins (review). Biointerphases.

[55] Biscop E (2019). Influence of cell type and culture medium on determining cancer selectivity of cold atmospheric plasma treatment. Cancers (Basel).

[56] Eggers B (2020). The beneficial effect of cold atmospheric plasma on parameters of molecules and cell function involved in wound healing in human osteoblast-like cells in vitro. Odontology.

[57] Girard F (2016). Formation of reactive nitrogen species including peroxynitrite in physiological buffer exposed to cold atmospheric plasma. R. Soc. Chem. Adv..

[58] Mitra S (2019). Impact of ROS generated by chemical, physical, and plasma techniques on cancer attenuation. Cancers.

[59] Park S-B (2015). Differential epigenetic effects of atmospheric cold plasma on MCF-7 and MDAMB-231 breast cancer cells. PLoS ONE.

[60] Xiang L, Xu X, Zhang S, Cai D, Dai X (2018). Cold atmospheric plasma conveys selectivity on triple negative breast cancer cells both in vitro and in vivo. Free Radical Biol. Med..

[61] Chao-Yu C (2018). Synergistic effects of plasma-activated medium and chemotherapeutic drugs in cancer treatment. J. Phys. D: Appl. Phys..

[62] Jalili A, Irani S, Mirfakhraie R (2016). Combination of cold atmospheric plasma and iron nanoparticles in breast cancer: Gene expression and apoptosis study. OncoTargets Ther..

[63] Li W (2019). Cold atmospheric plasma and iron oxide-based magnetic nanoparticles for synergetic lung cancer therapy. Free Radical Biol. Med..

[64] Adhikari M, Adhikari B, Ghimire B, Baboota S, Choi EH (2020). Cold atmospheric plasma and silymarin nanoemulsion activate autophagy in human melanoma cells. Int. J. Mol. Sci..

[65] Yu H (2018). Paclitaxel-loaded core–shell magnetic nanoparticles and cold atmospheric plasma inhibit non-small cell lung cancer growth. ACS Appl. Mater. Interfaces.

[66] Synowiec E, Hoser G, Bialkowska-Warzech J, Pawlowska E, Skorski T, Blasiak J (2015). Doxorubicin differentially induces apoptosis, expression of mitochondrial apoptosis-related genes, and mitochondrial potential in BCR-ABL1-expressing cells sensitive and resistant to Imatinib. BioMed Res. Int..

[67] Frisch SM, Francis H (1994). Disruption of epithelial cell-matrix interactions induces apoptosis. J. Cell Biol..

[68] Nomura Y, Tashiro H, Hisamatsu K (1989). In vitro clonogenic growth and metastatic potential of human operable breast cancer. Cancer Res..

[69] Markoutsa E (2014). Mono and dually decorated nanoliposomes for brain targeting, in vitro and in vivo studies. Pharm. Res..

[70] Kannavou M, Marazioti A, Stathopoulos GT, Antimisiaris SG (2021). Engineered versus hybrid cellular vesicles as efficient drug delivery systems: A comparative study with brain targeted vesicles. Drug Deliv. Transl. Res..

