# Supplementary material for: Synergistic effect of cold atmospheric pressure plasma and free or liposomal doxorubicin on melanoma cells
Source: Sci Rep. 2021 Jul 20;11:14788. doi: 10.1038/s41598-021-94130-7 (PMC8292331; doi:10.1038/s41598-021-94130-7)

Synergistic effect of Cold Atmospheric Pressure Plasma and Free or Liposomal Doxorubicin on melanoma cells

Konstantina Pefani-Antimisiari[1,&], Dimitrios K. Athanasopoulos[2,&], Antonia Marazioti[1,3,*], Kyriakos Sklias[2], Maria Rodi[4], Anne-Lise de Lastic[4], Athanasia Mouzaki[4], Panagiotis Svarnas[2,*], Sophia G. Antimisiaris[1,3]

Supplementary Information

Supplementary Data

Table S1. Analytical DLS results including the specific peaks measured in LIP and DOX-LIP samples. For each Peak the % Intensity is reported (in parenthesis).

**Sample**	**Mean hydrodynamic diameter (nm)**	**Peak 1 (%Intensity)**	**Peak 2 (%Intensity)**
Empty LIP	100.8 ± 2.6	107.15 (98.26)	4375 (1.73)
DOX-LIP	110.8 ± 2.6	114.13 (99.33)	1585 (0.66)

Figure S1. Viability (% of control) of B16F10 cells in the presence or absence of CAP (60s) and/or DOX (10μM) for 4h (similar conditions as the ones used in DOX uptake studies). As seen no toxicity was demonstrated by any of the treatments applied.

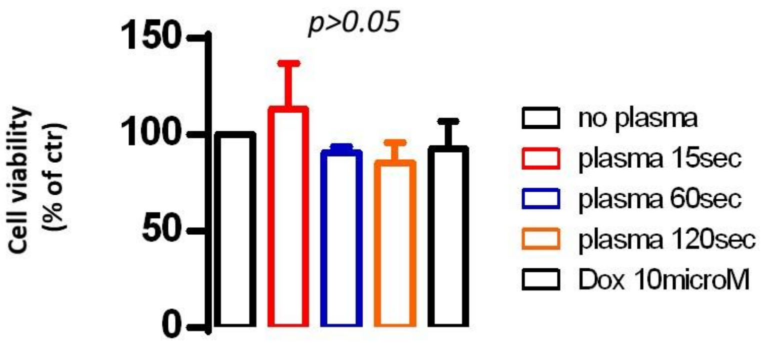

Figure S2. Trypan Blue Exclusion Assay in B16F10 cells following 4, 24 and 48h treatments with CAP (15s) and/or DOX (0.1μM). (a) Cell numbers; (b) numbers of Alive cells;

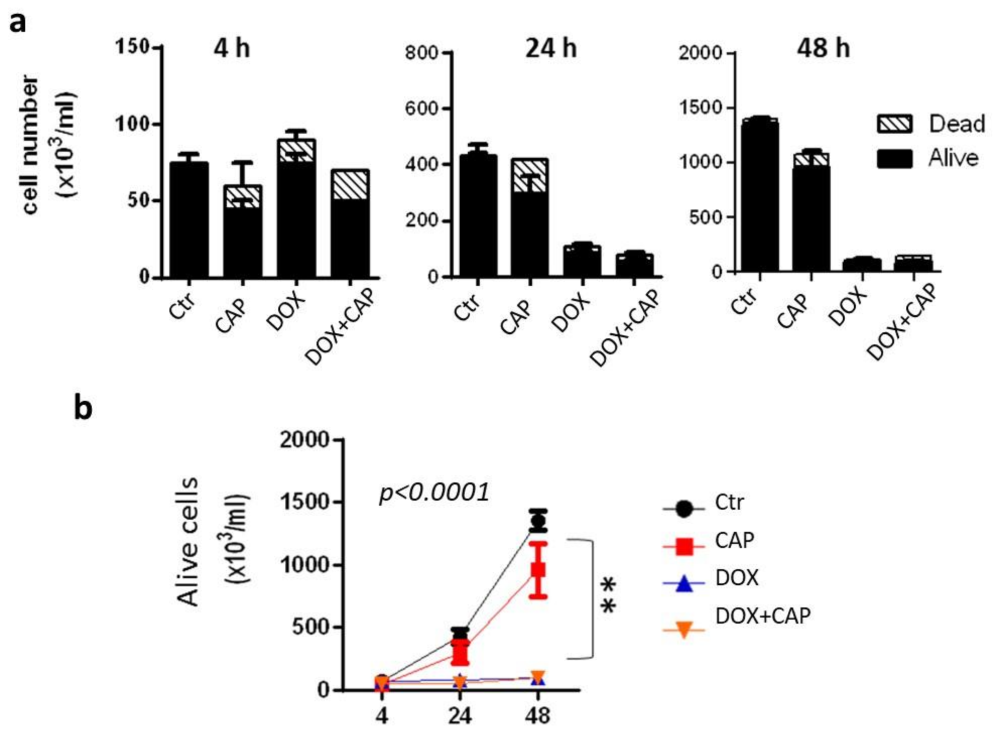

Supplement: Supplementary file 1 — Supplementary Information. [file 41598_2021_94130_MOESM1_ESM.pdf]
